# Investigating the effect of mesenchymal stem cells on the rate of clinical and pathological improvement of asthmatic lung in mouse model

**DOI:** 10.1016/j.reth.2023.12.013

**Published:** 2023-12-27

**Authors:** Kambiz Moghaddasi, Saeed Hesaraki, Farnoosh Arfaee, Seyyed Shamsadin Athari

**Affiliations:** a Department of Clinical Sciences, Science and Research Branch, Islamic Azad University, Tehran, Iran; b Department of Immunology, School of Medicine, Zanjan University of Medical Sciences, Zanjan, Iran

**Keywords:** Cell therapy, Regenerative medicine, Pulmonary disease

## Abstract

Asthma is a pulmonary disease and its pathophysiology includes inflammation, obstruction, edema of the airways, and mucus secretions in the airways. Mesenchymal stem cells (MSCs) are self-renewal that use the therapeutic potential of these cells can be applied as treatments of asthma. In this study, the effect of Mesenchyme stem cells on asthma was investigated.

MSCs were administrated for asthmatic mice and then, percentage of eosinophils in blood and bronchoalveolar lavage fluid (BALF), levels of interleukine (IL)-4 and Immunoglubolin (Ig)E were measured. Also histopathological study of lung tissue was done.

MSCs administration could control percentage of eosinophils in blood and BALF, levels of IgE and IL-4, eosinophilic inflammation, mucin realizing and goblet cell hyper-plasia.

Administration of MSCs as treatment of asthma can be a useful and applicable therapy in control of asthma symptoms.

## 1 Introduction

Asthma is one of the main diseases in world. The pathophysiology of asthma includes inflammation, obstruction, edema of the airways, mucus secretions in the airways that are involved in the obstruction and overreaction of the airways. There are degrees of mononuclear cell infiltration, eosinophilia, mucus secretion, and desquamation of the epithelium, smooth muscle hyperplasia, and finally structural changes in the airways or remodeling [[1], [2], [3]].

Overreaction of the airways to external and internal stimuli is seen in asthma. This is due to direct stimulation of smooth muscle cells around the airways and indirect stimulation by pharmacologically active substances secreted by mast cells and unmyelinated sensory neurons. A high degree of airway hyper responsiveness (AHR) is consistent with the severity of asthma symptoms, and for this reason, spirometry should be tested with airway responsiveness to bronchodilators, and also to exercise should be tested in exercise-induced bronchospasm. Receptors that are stimulated by stimuli and tension on the smooth muscles of the airways are cholinergic motor nerves [4,5]. T lymphocyte cells play an important role in regulation of airway inflammation, and unfortunately, current anti-inflammatory treatments in asthma do not seem to prevent the progression of the disease. Chronic inflammation of airways in some asthmatic patients causes changing in the structure of airways, remodeling and subepithelial fibrosis [3,5,6].

Based on the properties of self-renewal, regeneration and differentiation in physiological and experimental conditions, stem cells are able to create types of specific cells with specific actions. By definition, cell therapy is a subset of regenerative medicine, and isolation and application of stem cells has provided a powerful tool for research in biology. MSCs represent an extremely small population of powerful stem cells that the therapeutic potential of these cells are used. MSCs have the ability to be placed at the site of inflammation during tissue injuries, differentiate into different cell lines, and have immunomodulatory activities [7,8]. Also, stem cells have special characteristics such as self-renewal and differentiation into different cell lines, which make them an attractive method for cell therapy in the clinic and stem cell treatment is one of the emerging treatment strategies for several treatment-resistant diseases. However, due to many ethical and legal restrictions, clinical development and the progress of stem cell therapy has been relatively slow. Stem cells can activate the regulatory responses of the immune system and participate in the regulation of innate immunity and acquired immunity. They can also secrete various growth factors and cytokines to improve the lung environment and promote lung repair [7,9]. Considering that the main treatments of asthma in some cases have not been able to control the disease and often lead to relapses, and due to the lack of complete control, newer treatments are needed, so in this study, the effect of Mesenchyme stem cells in the rate of clinical and pathological improvement of asthmatic lung in mouse model was investigated.

## 2 Material and methods

### 2.1 Preparation and cultivation of stem cells

MSCs were extracted from the bone marrow of mice in the femur region by flushing method and after washing, they were confirmed by using specific markers. Then they grow and multiply in the cell culture medium. Finally, by counting and confirming the viability of the cells, which were used for administration to asthmatic mice [7,9].

## 3 Asthma model of animals under investigation

In this study, to model and develop allergic asthma in the relevant groups, 48 male Balb/c mice 8-week-old were divided in 4 groups and then subjected to sensitization and challenge by ovalbumin (OVA) protein until asthma symptoms in these animals appear. In briefly, first, on days 1 and 14, OVA was injected through the peritoneal cavity, and then on days 24, 26, 28, and 30, it is inhaled through intratracheal. Also, the cell treatment group was treated with MSCs on the 25th, 27th and 29th days according to the following grouping [3,7,9]. The groups included mice sensitized with OVA, mice sensitized with OVA and receiving MSCs, mice sensitized with OVA and receiving budesonide drug, and healthy mice that only received normal saline and did not receive OVA and it has not been sensitive. To investigate the effect of this treatment on asthmatic animals and compare them with control groups and healthy animals, the required samples were taken on the 31st day of study.

### 3.1 Counting of eosinophils in the blood of mice

After preparing the blood slide, the number of eosinophils and their percentage to the total cells were measured and counted.

### 3.2 Counting of eosinophils in bronchoalveolar lavage fluid of mice

First, BALF was obtained from the lungs of mice. Briefly, after anesthetizing the mice, the larynx area of the mice was opened and by making a small incision on the trachea, the tube for collecting the lavage liquid was directed into the trachea and then by sending PBS solution into the lungs, the liquid was collected again. After preparing slides with cytospin and staining with Gisma dye, the amount of these cells and their percentage in relation to the total cells were measured and counted.

### 3.3 Measurement of interleukin 4

In the BALF, the amount of the cytokine was measured with the cytokine (IL-4) measurement kit by enzyme-linked immunosorbent assay method.

### 3.4 Measurement of total IgE

In the collected blood, after separating the serum, amount of the total IgE was measured with the IgE measurement kit by ELISA method in the serum.

### 3.5 Preparation of histopathological section

Histopathological sections of lung tissue were prepared and stained with Haematoxylin and Eosin (H&E) and periodic acid Schiff (PAS) staining to investigate inflammation around airways, inflammation around vessels, hyperplasia of goblet cells and the amount of mucus secretion under a light microscope.

### 3.6 Statistical analysis

After data collection, data was entered into SPSS version 20 software and subjected to statistical analysis. The T-test was used to compare quantitative variables, and P value less than 0.05 was considered significant.

## 4 Result

### 4.1 Eosinophils in the blood

**Fig. 1:**
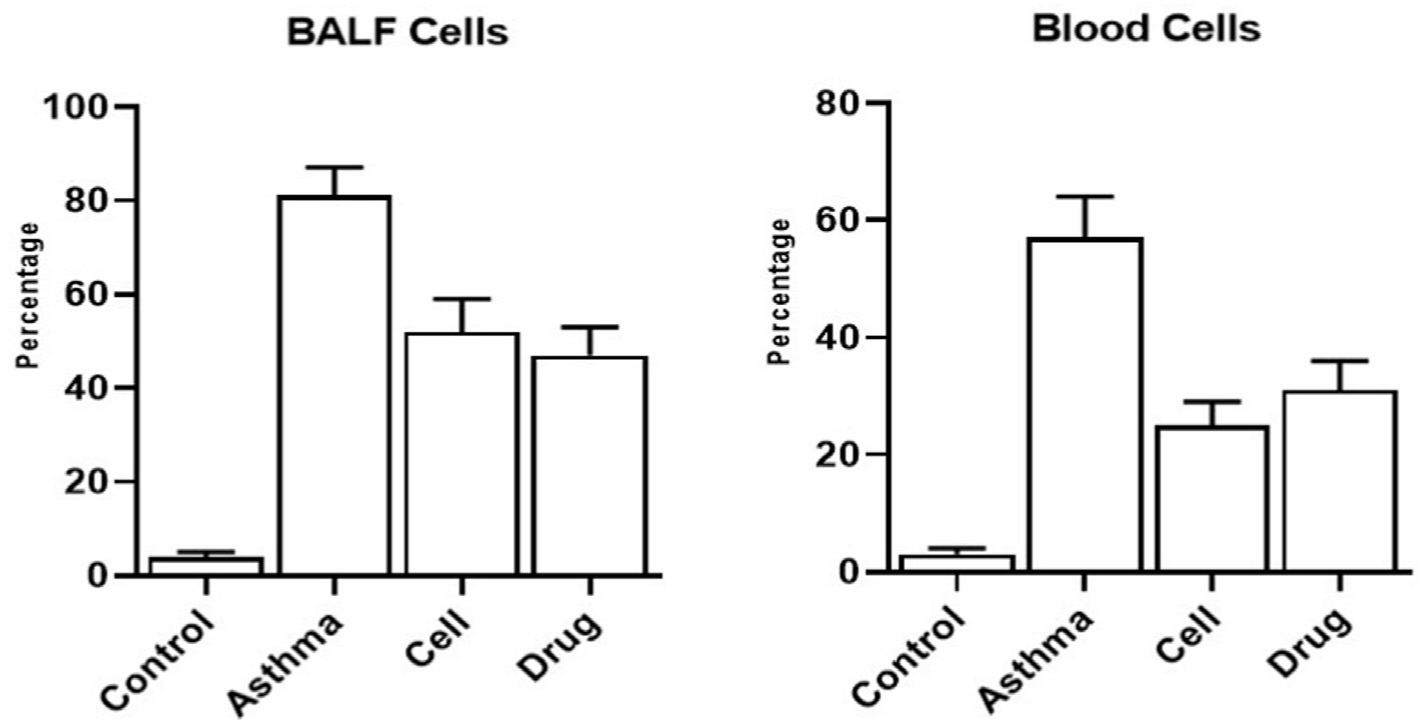
**Eosinophil's percentage.** Percentage of eosinophils were determined in blood and broncho-alveolar lavage fluid.

Percentage of eosinophil was enhanced in asthma (57 ± 7 %) group compare to healthy control group (3 ± 1 %). Cell therapy could control eosinophil's percentage (25 ± 4 %) in asthmatic mice (Fig. 1).

### 4.2 Eosinophils in the bronchoalveolar lavage fluid

Percentage of eosinophil in lung lavage was enhanced in asthma (81 ± 6 %) group compare to healthy control group (4 ± 1 %). Cell therapy could control eosinophil's percentage (52 ± 7 %) in asthmatic mice (Fig. 1).

### 4.3 IL-4

**Fig. 2:**
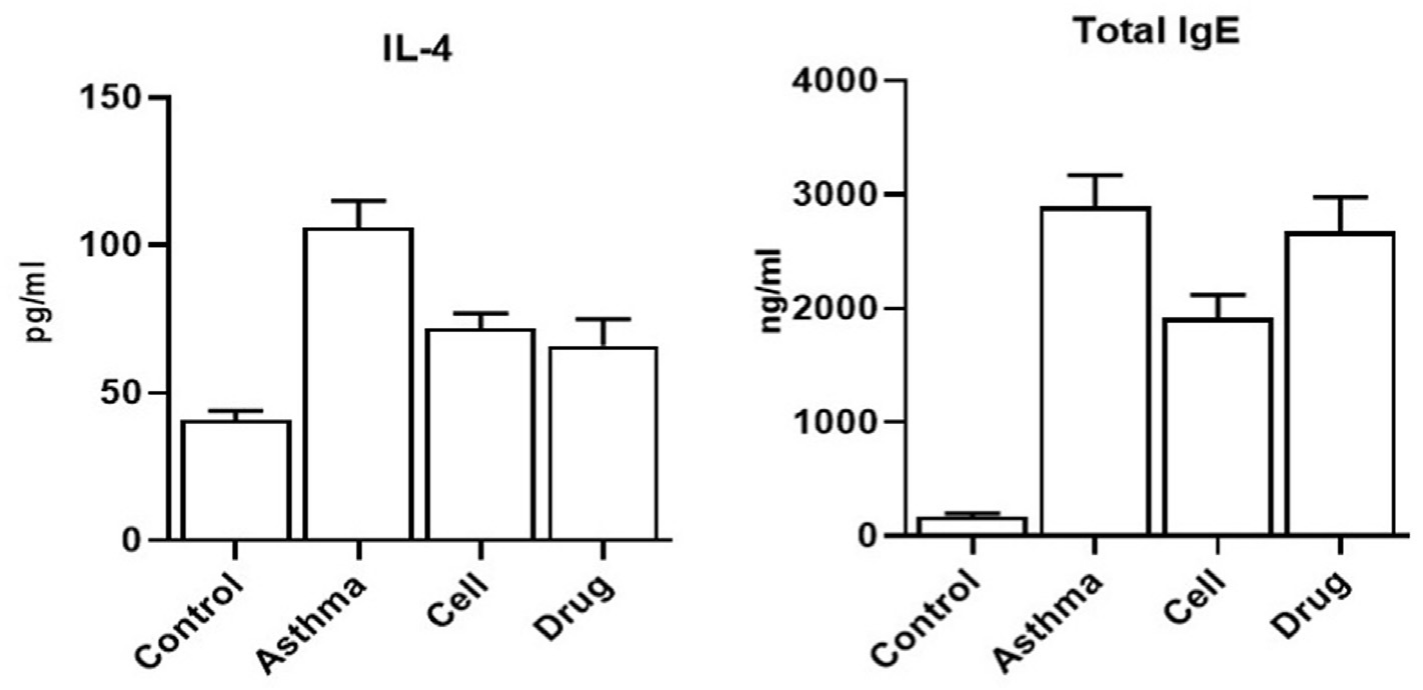
**Cytokine and Immunoglobulin.** Levels of IL-4 in broncho-alveolar lavage fluid. and IgE in blood were determined.

Amount of IL-4 was elevated in BALF of asthmatic mice (106 ± 9 pg/ml) compare to healthy control mice (41 ± 3 pg/ml). Cell therapy could control level of IL-4 (72 ± 5 pg/ml) in asthmatic mice (Fig. 2).

### 4.4 Total IgE

Amount of IgE was elevated in serum of asthmatic mice (2894 ± 274 ng/ml) compare to control mice (169 ± 28 ng/ml). Cell therapy could control level of IgE (1914 ± 204 ng/ml) in asthmatic mice (Fig. 2).

### 4.5 Histopathological section

**Fig. 3:**
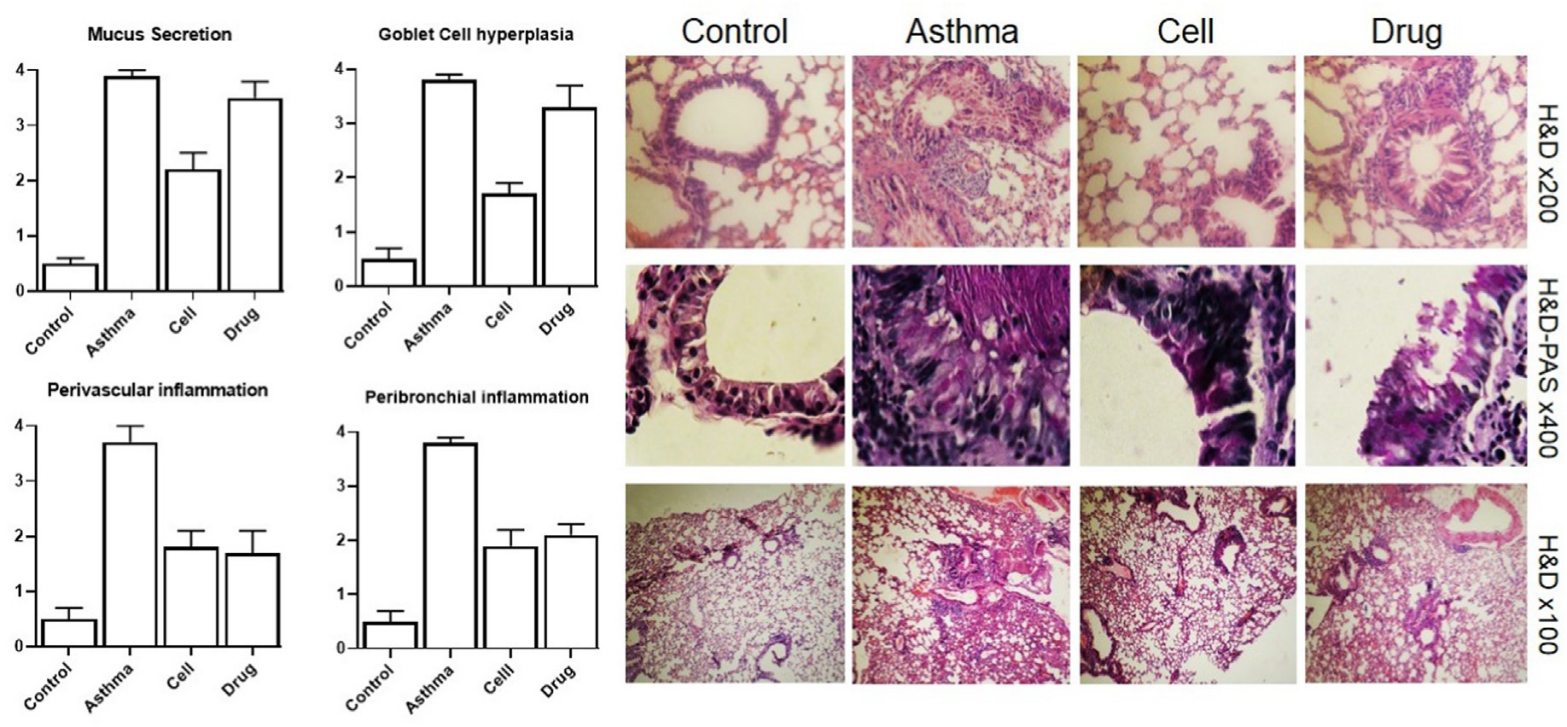
**Histopathology**. Inflammation around of airways and vessels, mucin realizing and goblet cell hyper-plasia were evaluated in lung section of all groups that were stained with H&E and H&E-PAS.

Inflammation (eosinophilic type) around bronchi and vessels were increased in asthma group (3.8 ± 1 and 3.7 ± 3 respectively) compare to healthy control group (0.5 ± 0.2 and 0.5 ± 0.2 respectively). Cell therapy could control Inflammation around bronchi (1.9 ± 0.3) and vessels (1.8 ± 0.3) in asthmatic mice (Fig. 3). Mucin realizing and goblet cell hyper-plasia were increased in asthma group (3.9 ± 1 and 3.8 ± 1 respectively) compare to healthy control group (0.5 ± 0.1 and 0.5 ± 0.2 respectively). Cell therapy could control mucin realizing (2.2 ± 0.3) and goblet cell hyper-plasia (1.7 ± 0.2) in asthmatic mice (Fig. 3).

## 5 Discussion

Asthma is a chronic inflammatory disease of the airways with changes in structure and function of the airways leading to non-specific excitability of the bronchi and obstruction. Asthma is one of the most common chronic diseases in western countries, and inflammation plays an essential role in the pathophysiology of asthma. Airway inflammation is associated with the reaction of various immune cells and mediators, which ultimately lead to changes in the pathophysiology of asthma. Bronchial inflammation and airway obstruction lead to clinical manifestations of cough, wheezing, and shortness of breath [10,11]. Inflammation in and around of the airways with an increase in the reaction of the bronchi, lead to spasms and characteristic symptoms of asthma, and in some chronic asthmatic patients, due to changes in the structure of the airways and remodeling, smooth muscle hyperplasia, increased vascular coiling in place, and subepithelial fibrosis, which causes reversibility of the airway obstruction, should not be seen. Airway inflammation is caused by the disruption balance of Th lymphocytes types (1 and 2), which type 2 of these lymphocytes secrete IL-4, IL-5, IL-9 and IL-13. On the other hand, the mucus plug may take several weeks to disappear, and the remodeling of the airways is caused by chronic inflammation and is effective on the reversibility of the airways [3,6,10,11].

Undoubtedly, one of the most important advantages of mesenchymal stem cells is the use of the therapeutic potential of these cells Mesenchymal stem cells are pluripotent cells that were initially identified in the mesoderm and ectoderm layers of early embryonic stages and express specific cell surface markers such as cluster of differentiation (CD)90, CD73, and CD105, while they are negative for CD31, CD45, and CD34 markers. Mesenchymal stem cells were first isolated from bone marrow, but then they were discovered in different tissues such as fat, dental pulp, umbilical cord and placenta [12]. MSCs express a small level of human leukocyte antigen class I molecules and do not express leukocyte class II molecules or stimulatory molecules, and this characteristic allows stem cells to escape from the defensive reactions of B, T and natural killer (NK) cells [13]. Mesenchymal stem cells have been widely used in inflammatory diseases related to the immune system, such as acute respiratory syndromes [14]. In the current study, amount of IL-4 in bronchoalveolar lavage fluid and amount of total IgE in serum of asthmatic mice were elevated. Cell therapy with MSCs could control level of IgE and IL-4 in serum and bronchoalveolar lavage fluid (respectively) of asthmatic mice.

Mesenchymal stem cells can cause cell proliferation and repair tissue damage by secreting Hepatocyte Growth Factor (HGF), Vascular Endothelial Growth Factor (VEGF), Keratinocyte Growth Factor (KG) and Fibroblast Growth Factor (FGF) [15]. Secreted KGF, HGF and angiopoietin 1 by stem cells have pro-angiogenic, anti-inflammatory and proliferative effects and lead to the reduction of apoptosis of alveolar epithelial cells and endothelial cells. MSCs are also able to secrete HGF through extracellular vesicles, thereby reduce inflammatory damage and increase autophagy. In addition, MSCs can reduce the level of profibrotic factors to improve the microenvironment of lung cells and prevent pulmonary fibrosis, especially in patients with respiratory diseases. A possible mechanism for this effect is Exosome regulation [16,17]. By reducing the levels of transforming growth factor-beta (TGF-β) and tumor necrosis factor-alpha (TNF-α), collagen type I, collagen type III, hydroxyproline and serum ceruloplasmin in lung tissues, MSC exosomes can regenerate epithelial and alveolar cells, reduce alveolar inflammation and inhibit the apoptosis of endothelial cells, as a result of the reduction of pulmonary fibrosis, and inflammation [18]. In our study, it was observed that percentage of eosinophils was enhanced in blood and bronchoalveolar lavage fluid of asthma group and cell therapy with MSCs could control eosinophil's percentage in blood and bronchoalveolar lavage fluid of asthmatic mice.

In a study conducted by Castro et al., in 2019 under the title of the effect of multiple injections of mesenchymal stromal cells in asthma caused by occupational factors. In this study, house dust mite was used intranasally in mice, then multiple injections of mesenchymal stromal cells were performed intravenously, multiple injections were accompanied by a decrease in lung inflammation, a decrease in interleukin 4, 13, total leukocyte and lymphocyte TCD4, and the number of eosinophils in Bronchoalveolar lavage. Also, total leukocyte count were decreased in the bone marrow, spleen and mediastinal lymph nodes, and the injection of multiple doses of mesenchymal stromal cells also reduced the production of collagen fibers and also changed the level of TGF-β factor, also the injection of three doses related to the better effect, further reduced the inflammatory parameters as well as the reduction of alpha-actin filaments in the lung tissue. The injection of two doses and more was associated with an increase in galectin levels, but only the injection of three doses and higher was associated with the increase of CD39+ cells in the thymus. Finally, multiple injections of mesenchymal stromal cells were associated with reducing inflammation and remodeling [19]. MSCs activate T regulatory cells (T-reg) that cause the production of anti-inflammatory cytokines such as interleukin 10 and TGF-β, which suppress airway inflammation. In addition, these cells regulate the function of macrophages and dendritic cells, which are effective cells in asthma. Stromal cells also change the phenotype of macrophages from M1 to M2, which weakens asthma. Finally, they suppress the proliferation of smooth muscle cells in the airways and the secretion of mucus from goblet cells. As a result, it leads to the improvement of clinical and pathological symptoms in the lungs of asthmatics [20]. In a study conducted by Neza Adamik et al., in 2022 under the title of the effect of mesenchymal stem cells derived from autologous adipose tissue in the treatment of severe asthma in horses. The results indicated that the use of mesenchymal stromal cells associated with the reduction of interleukin 17, TNF-α, β1 and had a long-term positive effect on severe asthma in horses [21]. In a study conducted by Shin et al., in 2021 under the title of the effect of mesenchymal stem cells in the treatment of severe asthma by means of T helper2 cells, the effect of mesenchymal stromal cells derived from human umbilical cord blood was performed on two mouse models with severe asthma, and the results indicated that these cells improve asthma by suppressing asthmagenic cytokines [20]. In a study conducted by Abreu et al., in 2018 under the title of the effect of mesenchymal stromal cells on environmental allergic asthma, it was shown that mesenchymal stromal cells have a good potential to reduce inflammation and remodeling in cases of asthma that was caused by occupational exposure [22]. In the current study, increased eosinophilic type of inflammation around bronchi and vessels were in asthma group were controlled by MSCs therapy. Also, mucin realizing and goblet cell hyper-plasia were decreased in asthma group by MSCs therapy. It was observed that MSCs therapy could control pathophysiology of asthma and administration of MSCs as treatment of asthma can be a useful and applicable therapy.

## Ethics approval and consent to participate

The study was approved by the ethic committee of Science and Research Branch, Islamic Azad University (IR.IAU.SRB.REC.1402.136).

## Consent for publication

Not Applicable.

## Availability of data and materials

Not Applicable.

## Funding

Not Applicable.

## Authors' contributions

KM, SH, FA, and SSA, participated in the planning of project, study, testing, and drafting of the manuscript. The study is DVSc thesis of KM. FA and SSA are supervisors and SH is co-supervisor.

## Declaration of competing interest

The authors declare that they have no known competing financial interests or personal relationships that could have appeared to influence the work reported in this paper.
